# Molecular Characterization and Preliminary NGS Profiling of Terbinafine-Resistant *Trichophyton indotineae* Isolates in Italy

**DOI:** 10.3390/pathogens15040435

**Published:** 2026-04-17

**Authors:** Deborah Cruciani, Manuela Papini, Luigi Pisano, Roberta Calcaterra, Donatella Pietrella, Tommaso Galeotti, Paolo Fazii, Antonia Meloscia, Martina Torricelli, Marco Di Domenico, Alessandro Fiorucci, Sara Spina, Silvia Crotti

**Affiliations:** 1 Istituto Zooprofilattico Sperimentale dell’Umbria e delle Marche “Togo Rosati”, 06126 Perugia, Italy; m.torricelli@izsum.it (M.T.); a.fiorucci@izsum.it (A.F.); s.spina@izsum.it (S.S.); s.crotti@izsum.it (S.C.); 2 Clinica Dermatologica di Terni, Dipartimento di Medicina e Chirurgia, Università degli Studi di Perugia, 06123 Perugia, Italy; manuelapapini@tiscali.it; 3 Section of Dermatology, Health Sciences Department, University of Florence, 50122 Florence, Italy; luigi.pisano88@yahoo.it; 4 National Institute for Health, Migration and Poverty, 00153 Rome, Italy; roberta.calcaterra@inmp.it; 5 Microbiology Unit, Perugia General Hospital, 06100 Perugia, Italy; donatella.pietrella@ospedale.perugia.it; 6 Dermatology Section, Department of Medicine and Surgery, University of Perugia, 06100 Perugia, Italy; dr.tommasogaleotti@gmail.com; 7 Microbiologia e Virologia Clinica Section, Presidio Ospedaliero “Spirito Santo”, 65123 Pescara, Italy; paolo.fazii@asl.pe.it (P.F.); antonia.meloscia@asl.pe.it (A.M.); 8 Istituto Zooprofilattico Sperimentale dell’Abruzzo e del Molise “Giuseppe Caporale”, Campo Boario, 64100 Teramo, Italy; m.didomenico@izs.it

**Keywords:** dermatophytosis, NGS, *SQLE*, squalene epoxidase, terbinafine resistance, *tinea*, *Trichophyton indotineae*

## Abstract

*Trichophyton indotineae* is an emerging dermatophyte associated with extensive, chronic, recalcitrant, and frequently terbinafine-resistant dermatophytosis worldwide. In this study, 30 *T. indotineae* strains isolated in Italy were investigated. The isolates were obtained from patients originating from Asian countries, from patients from other countries, and from Italian patients who reported no travel outside Italy in the preceding years. Clinical isolates were identified by internal transcribed spacer (ITS) sequencing and analyzed to assess the occurrence and molecular basis of terbinafine resistance. Terbinafine resistance was detected in 18 strains (60%) using a real-time PCR assay. Sequencing of the squalene epoxidase (*SQLE*) gene revealed mutations associated with resistance, including L393S in nine strains and F397L in another nine strains. NGS analysis confirmed two terbinafine-resistant strains carrying the L393S and F397L mutations, respectively, and detected the A448T mutation in one terbinafine-susceptible strain. These findings demonstrate the presence of terbinafine-resistant *T. indotineae* across five regions of Italy and confirm the occurrence of *SQLE* mutations previously linked to antifungal resistance. Data obtained also support a link with endemic Asian areas, other than suggesting the possible occurrence of autochthonous transmission in Italy.

## 1. Introduction

*Trichophyton indotineae* (formerly *T. mentagrophytes* genotype VIII) is a recently recognized, globally emerging dermatophyte species [1]. It was first described on the Indian subcontinent, where it displaced *T. rubrum* in prevalence, reflecting a notable epidemiological shift [2]. Infections caused by *T. indotineae* typically present as extensive, intensely pruritic, erythematous and scaly maculopapular lesions, with an active, well-defined border and centrifugal expansion, evolving into erythematous infiltrated plaques, particularly in long-standing cases. The lesions are often annular or polycyclic and may involve large body areas, frequently accompanied by marked inflammation and a burning sensation. Multifocal involvement of different body sites is common, and the clinical course is often chronic or recalcitrant to conventional antifungal therapy with terbinafine. *Tinea corporis*, *tinea cruris*, and *tinea faciei* represent the most common clinical manifestations, whereas nail and scalp involvement is relatively uncommon. Additionally, *T. indotineae* exhibits high rates of terbinafine resistance. Terbinafine is an allylamine antifungal that inhibits the squalene epoxidase enzyme (SQLE) in the early stages of ergosterol biosynthesis; this results in toxic intracellular accumulation of squalene and depletion of ergosterol in the fungal cell membrane, thereby compromising membrane integrity and function, leading to fungal cell death. Various mechanisms linked to transcriptional modifications may underlie terbinafine resistance, including single nucleotide variants (SNVs) in the *SQLE* gene that result in amino acid substitutions inducing conformational changes in the squalene epoxidase enzyme. According to Gupta et al., F397L is the most frequent substitution in terbinafine-resistant *T. indotineae* (33%) strains, followed by F397L + A448T combination (18.9%), and L393S (13.7%) [3]. The single A448T mutation has been identified in several *T. indotineae* isolates (24.5%) even if its distal position to the terbinafine binding pocket does not typically confer resistance to terbinafine; therefore, A448T has been associated with a decreased susceptibility to azole antifungals [4]. These findings suggest that the impact of mutations on resistance is dependent on their structural proximity to the enzyme’s binding site, where alterations that diminish terbinafine binding affinity can facilitate fungal survival [3]. Less common mutations linked to terbinafine resistance include Q408L, F397I, S395P, F415C, and other combinations [5]. Furthermore, a minority of terbinafine-resistant isolates may lack known *SQLE* mutations, implying possible alternative resistance mechanisms (e.g., efflux pumps overexpression and other adaptive cellular responses), although *SQLE* substitutions remain the primary known mechanism [3].

In Italy, *T. indotineae* isolates have been detected in patients originally from Asian countries such as India, Bangladesh, and Pakistan, as well as in Italian patients reporting contact with persons from or travel to these regions. Notably, some Italian patients denied any travel outside Italy in the preceding years, hypothesizing an autochthonous transmission. Among the terbinafine-resistant isolates, the mutations identified included F397L, F397L + A448T, L393S, and F415C [6,7,8].

Several authors concur that molecular techniques are indispensable for reliably differentiating *T. indotineae* from other members of the *T. mentagrophytes*/*T. interdigitale* species complex and for providing additional strain-level information, including SNVs associated with antifungal resistance [3,9,10,11]. A good diagnostic practice is essential to guide selection of the most appropriate antifungal therapy, thereby improving clinical outcomes and reducing the likelihood of recurrence and further dissemination.

A positive trend is the marked increase in publications on *T. indotineae* in recent years, reflecting growing awareness of its diagnosis and clinical management. Furthermore, *T. indotineae* outbreaks in dogs have been reported in India [12], Iran [13], and Egypt [14] suggesting a host range extension from humans to animals and raising concerns about zoonotic transmission.

In this study, *T. indotineae* isolates were identified and genetically analyzed using PCR and Sanger sequencing to determine their susceptibility or resistance to terbinafine. A subset of samples was additionally analyzed using next-generation sequencing (NGS) to evaluate an alternative sequencing strategy and to minimize potential operator-dependent errors associated with Sanger sequence processing. The resulting data were useful in optimizing antifungal therapy and improving the knowledge about the distribution of *T. indotineae*. Although the number of isolates is limited to support conclusions regarding geographical distribution, this study represents the first to investigate a broader area of Italy, covering five regions.

## 2. Materials and Methods

### 2.1. Study Population

**Figure 1:**
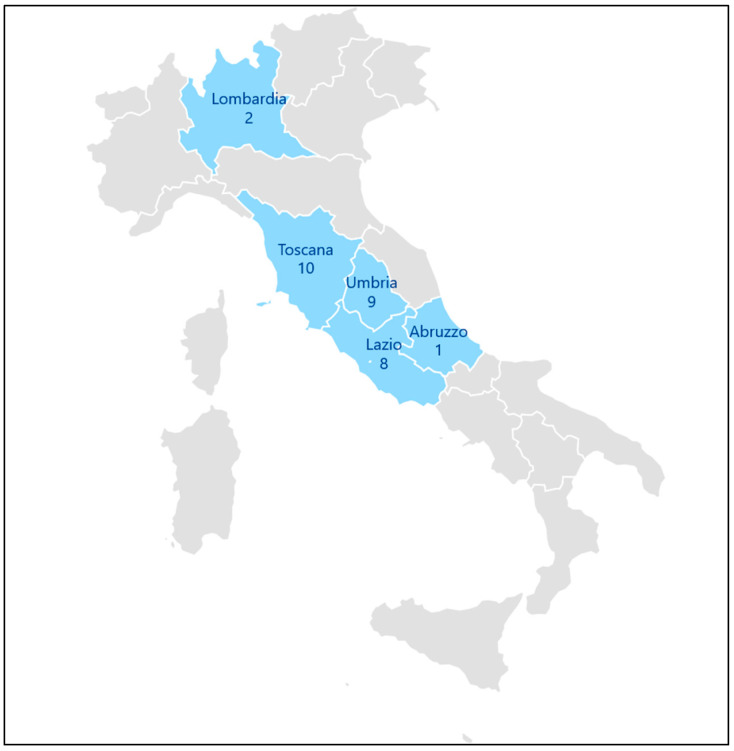
Geographic distribution of the medical centers involved in the study across central–northern Italy regions.

Since June 2023 to December 2025, a total of 30 *T. indotineae* strains were isolated from human samples in six different public medical centers and one private medical clinic. These health care facilities were located in five regions of central–northern Italy: Umbria (Santa Maria Hospital in Terni and Santa Maria della Misericordia Hospital in Perugia), Toscana (Piero Palagi Hospital in Florence), Lazio (National Institute for Health, Migration and Poverty in Rome), Lombardia (Papa Giovanni XXIII Hospital and the private medical clinic in Bergamo), and Abruzzo (Santo Spirito Hospital in Pescara) (Figure 1).

Patients, 21 male (70%) and 9 female (30%), with an average age of 36 (range: 11–60 years old), originated from Bangladesh (n = 16), Italy (n = 5), India (n = 3), Peru (n = 2), Sri Lanka (n = 1), Philippines (n = 1), Romania (n = 1), and Nepal (n = 1). They underwent clinical examination showing dermatological skin or nail lesions listed in Table 1 and attributable to dermatophyte fungal infection. The majority of patients presented with a single clinical manifestation: *tinea corporis* (n = 12, 40%), *tinea cruris* (n = 2, 6.7%), *tinea pedis* (n = 2, 6.7%), and *tinea faciei* (n = 1, 3.3%). Twelve patients exhibited two concurrent clinical manifestations: *tinea corporis* + *tinea cruris* (n = 8, 26.7%), *tinea corporis* + onychomycosis (n = 2, 6.7%), *tinea cruris* + *tinea pedis* (n = 2, 6.7%). In one case (3.3%) a combination of three clinical manifestations—*tinea corporis* + *tinea cruris* + *tinea faciei*—was observed.

### 2.2. Sample Collection

During the clinical examination, direct light microscopy after 20% KOH was performed to detect the presence of fungal filaments. Skin scraping and nail clipping samples were then collected by the dermatologist, inoculated onto Dermasel agar, incubated at 25 ± 1 °C, and observed daily. Fungal colonies grew over the course of a week: they were isolated and evaluated based on their macroscopic and microscopic features.

### 2.3. Molecular Analysis for Dermatophyte Species Identification

Polymerase Chain Reaction (PCR) and Sanger sequencing were performed at the Istituto Zooprofilattico Sperimentale of Umbria and Marche regions “Togo Rosati” (IZSUM) as previously described [9,15]. *Trichophyton mentagrophytes* var. *mentagrophytes*, *T. mentagrophytes* var. *interdigitale*, and *T. mentagrophytes* var. *indotineae* reference sequences were used to align the consensus sequences obtained for species identification [16].

### 2.4. Terbinafine Susceptibility

The DermaGenius^®^ Resistance Multiplex real-time PCR assay (Pathonostics^®^, Maastricht, The Netherlands) was employed to differentiate mutant and wild-type profiles associated with terbinafine resistance and susceptibility, respectively. Terbinafine behavior was investigated by analyzing the melting temperature (T_m_): a T_m_ between 54.0 °C and 64.0 °C was indicative of terbinafine-resistant strains harboring mutant *SQLE*, whereas a T_m_ between 64.5 °C and 68.0 °C corresponded to terbinafine-susceptible strains with wild-type *SQLE*. Each melting peak corresponded to a specific amino acid substitution at position 393 or 397 of the *SQLE* gene (e.g., F397L, L393F, L393S, F397I, or F397V); however, this assay does not precisely identify the exact nucleotide substitution within *SQLE*. Therefore, an additional end-point PCR was performed to amplify the gene encoding squalene epoxidase, using primers Tricho SE-F0 (5′-TGTAAAACGACGGCCAGTTGACAGCGACAAGTGCCA-3′) and TINT SE-R0 (5′-CAGGAAACAGCTATGACCAAAGAGCTAGAGATAAGCCTATCTG-3′) [17]. PCR was carried out in a 50 μL reaction volume and the conditions included initial denaturation for 5 min at 95 °C followed by 34 cycles of 30 s at 95 °C, 30 s at 60 °C, and 180 s at 72 °C [18]. The amplicon of about 1500 bp was then sequenced using internal primers TRI-SE-F3 (5′-TGTAAAACGACGGCCAGTGGAATATCTCCCCATACAACCAG-3′) and TRI-SE-R3 (5′-CAGGAAACAGCTATGACCCCTCCCTTCTCCAACGCAG-3′) [17]. Consensus nucleotide sequences were then translated into amino acid sequences and subsequently compared to the wild-type squalene epoxidase reference protein QVD37574 retrieved from the NCBI Protein database to assess sequence variation.

### 2.5. Next Generation Sequencing

To explore an alternative sequencing approach, three samples were preliminarily selected and subjected to next-generation sequencing (NGS) at the National Reference Centre for Whole Genome Sequencing of microbial pathogens (IZSAM, Teramo, Italy). The same *SQLE* PCR product generated by Tricho SE-F0 and TINT SE-R0 also represented the input for NGS library preparation using the Illumina DNA Prep kit. Sequencing was then performed on an Illumina NextSeq 1000 instrument (Illumina, San Diego, CA, USA), employing NextSeq 1000/2000 P1 XLEAP-SBS Reagents and a 600-cycle (2 × 300 bp) P1 cartridge.

Raw reads (FastQ) were pre-processed using Fastp v0.23.4 [19]. The filtered reads were then mapped against the 1439 bp reference sequence of the *T. indotineae* squalene epoxidase mRNA (voucher 216520/17; GenBank accession MW187977.1) [6,7,20]. Variant calling, including single nucleotide polymorphisms (SNPs) and insertions/deletions (INDELs), was performed using Snippy v4.6.0 (https://github.com/tseemann/snippy, accessed on 16 March 2026) to generate comprehensive Variant Call Format (VCF) reports. Data analysis was performed using the GenPat Platformversion 26.04.1 (https://genpat.izs.it, accessed on 16 March 2026), a bioinformatics suite developed by the Italian National Reference Centre for Whole Genome Sequencing of Microbial Pathogens. Finally, the same workflow was used to test a reduced subsample of reads (100 K per sample) using Rasusa [21] in order to further reduce the cost of sequencing.

**Table 1:** Information on the *T. indotineae*-affected patients and the sequences deposited in the GenBank database.

Case	Year	Sex	Age	Native Country	Clinical Manifestation	Accession Number ITS	Reference ITS	Terbinafine S/R ^1^	*SQLE* Mutation	Accession Number *SQLE*	Reference *SQLE*
1	2023	F	42	India	*Tinea corporis* and onychomycosis	OR192943	[22]	S	/	/	This study
2	2023	F	33	Sri Lanka	*Tinea corporis* and onychomycosis	OR880561	[15]	R	F397L	PZ050527	This study
3	2024	F	44	Peru	*Tinea pedis*	PP898430	[15]	S	/	/	This study
4	2024	M	16	Peru	*Tinea pedis*	PP898431	[15]	S	/	/	This study
5	2024	M	28	Bangladesh	*Tinea corporis*	PP898432	[15]	R	L393S	PZ050528	This study
6	2024	M	38	Bangladesh	*Tinea corporis*	PP898433	[15]	R	L393S	PZ050529	This study
7	2024	M	47	Bangladesh	*Tinea cruris*	PP898434	[15]	S	/	/	This study
8	2024	F	39	Bangladesh	*Tinea corporis*	PP898435	[15]	R	L393S	PZ050530	This study
9	2024	M	20	Bangladesh	*Tinea faciei*	PP898436	[15]	R	L393S	PZ050531	This study
10	2024	M	38	Bangladesh	*Tinea corporis*	PP898437	[15]	S	/	/	This study
11	2024	M	28	Bangladesh	*Tinea corporis* and *tinea cruris*	PP898438	[15]	R	F397L	PZ050532	This study
12	2024	M	37	Italy	*Tinea corporis* and *tinea cruris*	PP898439	[15]	R	L393S	PZ050533	This study
13	2024	F	22	India	*Tinea corporis* and *tinea cruris*	PQ892291	This study	R	F397L	PZ050534	This study
14	2024	F	19	Philippines	*Tinea corporis, tinea cruris*, and *tinea faciei*	PQ892292	This study	S	/	/	This study
15	2024	M	39	Italy	*Tinea cruris* and *tinea pedis*	PQ892293	This study	R	L393S	PZ050535	This study
16	2024	M	46	Bangladesh	*Tinea corporis*	PQ892294	This study	R	F397L	PZ050536	This study
17	2024	F	20	Romania	*Tinea corporis* and *tinea cruris*	PQ892295	This study	R	L393S	PZ050537	This study
18	2025	M	53	Italy	*Tinea corporis*	PZ028454	This study	R	L393S	PZ050538	This study
19	2025	M	29	Bangladesh	*Tinea corporis*	PZ028455	This study	S	/	/	This study
20	2025	M	36	Italy	*Tinea cruris*	PZ028456	This study	R	F397L	PZ050539	This study
21	2025	M	60	Italy	*Tinea corporis*	PZ028457	This study	S	/	/	This study
22	2025	F	15	Bangladesh	*Tinea corporis*	PZ028458	This study	S	/	/	This study
23	2025	M	29	Bangladesh	*Tinea corporis*	PZ028459	This study	R	F397L	PZ050540	This study
24	2025	M	36	Bangladesh	*Tinea corporis*	PZ028460	This study	S	(A448T) *	/	This study
25	2025	M	25	Bangladesh	*Tinea corporis* and *tinea cruris*	PZ028461	This study	S	/	/	This study
26	2025	M	11	India	*Tinea corporis*	PZ028462	This study	S	/	/	This study
27	2025	F	13	Bangladesh	*Tinea corporis* and *tinea cruris*	PZ028463	This study	R	F397L	PZ050541	This study
28	2025	M	18	Bangladesh	*Tinea corporis* and *tinea cruris*	PZ028464	This study	R	L393S	PZ050542	This study
29	2025	M	15	Nepal	*Tinea cruris* and *tinea pedis*	PZ028465	This study	R	F397L	PZ050543	This study
30	2025	M	18	Bangladesh	*Tinea corporis* and *tinea cruris*	PZ028466	This study	R	F397L	PZ050544	This study

^1^ S: terbinafine susceptible; R: terbinafine resistant. * (A448T): mutation found through NGS; NGS was only performed in two terbinafine-resistant cases (18 and 23) and in susceptible case 24.

## 3. Results

### 3.1. Fungal Culture

Mycological examination allowed us to isolate 30 fungal colonies attributable to *Trichophyton* spp. Macroscopically, the colonies appeared flat and powdery to granular. Their surface was white to cream-colored, with occasional beige to light brown hues developing centrally as growth progressed. Reverse pigmentation of the agar exhibited light yellowish to yellow-brown tones. Microscopically, hyaline and septate hyphae were observed, together with several hyaline and smooth-walled microconidia showing spherical to subspherical and occasional clavate to pyriform shapes. Macroconidia were observed very rarely: when present, they were smooth, thin-walled, and multicellular, and clavate in shape.

As many authors agree, the phenotypic features observed overlap extensively between dermatophytes belonging to the *T. mentagrophytes*/*T. interdigitale* species complex; therefore, molecular analyses become necessary for species identification and terbinafine resistance characterization [3,9,10,11].

### 3.2. Isolate Molecular Identification

According to Gupta et al. [16], ITS sequencing and comparison of the resulting consensus sequences with reference sequences for *T. mentagrophytes* var. *mentagrophytes*, *T. mentagrophytes* var. *interdigitale*, and *T. mentagrophytes* var. *indotineae* highlighted nucleotide substitutions at positions 125 and 462, allowing the identification of all 30 fungal strains as *T. indotineae*.

### 3.3. Terbinafine Susceptibility

**Figure 2:**
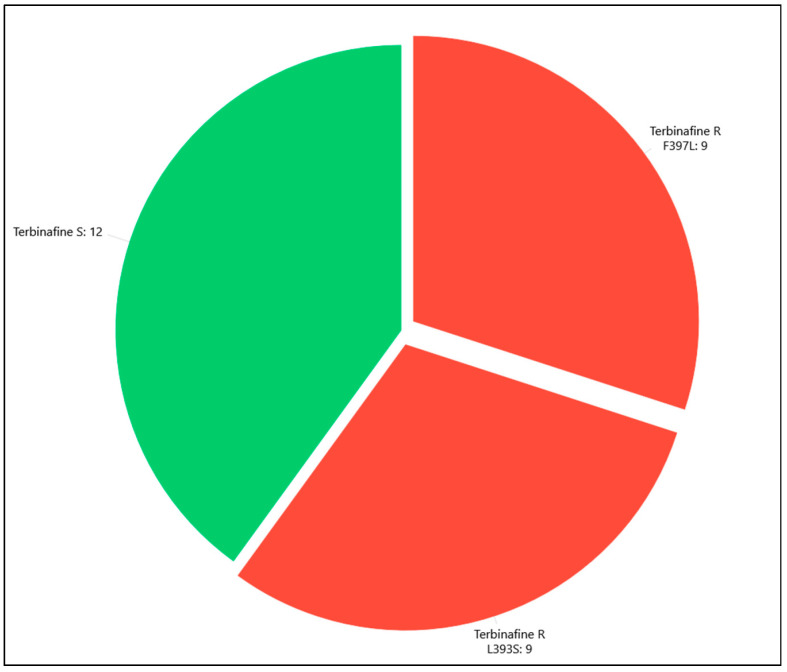
*T. indotineae* terbinafine-susceptible strains (in green) and terbinafine-resistant strains (in red). F397L and L393S substitutions are shown in detail.

The DermaGenius^®^ Resistance Multiplex real-time PCR assay identified 18 terbinafine-resistant strains (60%) and 12 terbinafine-susceptible strains (40%) (Table 1). Among the 18 resistant strains, L393S and F397L substitutions were each detected in nine (50%) cases (Figure 2).

**Figure 3:**
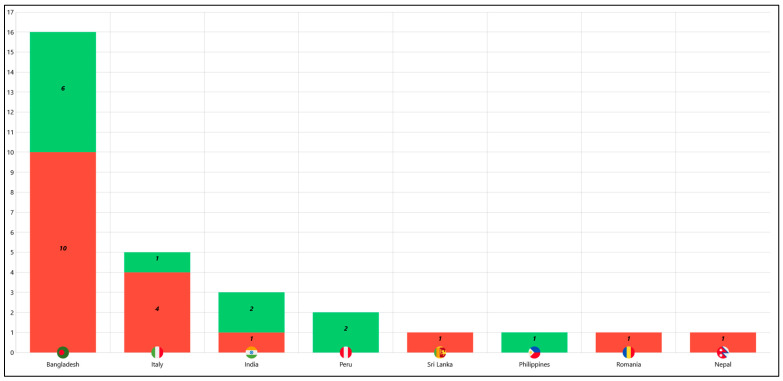
*T. indotineae* strains correlated to patients’ country of origin. In red are terbinafine-resistant strains and in green are terbinafine-susceptible strains.

**Figure 4:**
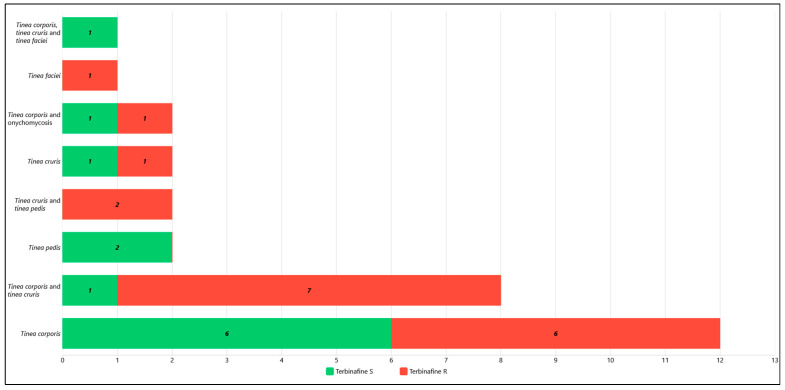
*T. indotineae* strains correlated to patients’ clinical manifestations. In red are terbinafine-resistant strains and in green are terbinafine-susceptible strains.

The resistance patterns of the *T. indotineae* strains are presented in Figure 3 and Figure 4, correlating them with the patients’ country of origin and their clinical manifestations, respectively.

The sequences obtained from the ITS and *SQLE* sequencing were deposited in the GenBank database under the accession number listed in Table 1.

### 3.4. Next Generation Sequencing

Sequencing yielded a total of 3.6 million reads with a mean Phred quality score (Q-score) 37.5. The VCF report confirmed 100% reference coverage (1439/1439), extending through both distal ends where sequencing depth typically declines. Even in these regions, the mean minimum coverage was maintained at 5800X. Two terbinafine-resistant strains (cases 18 and 23) each harbored a SNP at positions 1178 (T→C) and 1189 (T→C), respectively, consistent with the Sanger sequencing results and corresponding to the L393S and F397L substitutions. Case 24 showed a SNP at position 1342 (G→A), corresponding to the amino acid substitution A448T. Given this isolate’s susceptibility to terbinafine, Sanger sequencing analysis was not performed.

The same SNP results were obtained using 100K reads per sample with a mean minimum coverage in both distal ends 284X (lowest 271X; highest 293X).

## 4. Discussion

In this study, 30 *T. indotineae* strains were investigated to assess the occurrence and molecular basis of terbinafine resistance. More than two-thirds of the strains were isolated from patients originally from endemic regions (India, Sri Lanka, Bangladesh, Philippines, and Nepal). Some other patients with a history of contact with individuals from or travel to these regions were also included, corroborating the epidemiological links of *T. indotineae* to Asian endemic areas, as confirmed in other studies [10,23]. Moreover, some Italian patients denied any travel outside Italy in the preceding years, suggesting the possibility of autochthonous transmission in Italy.

Patients included in this study were from five regions of central–northern Italy, reflecting active multidisciplinary collaboration among different professionals and public and private institutions within a One Health framework.

The higher prevalence among male patients (70%) and a median age of 36 years closely mirrored the findings of a genomic study by Ribeiro dos Santos et al. [5], indicating a strong concordance between the demographic profiles.

In this investigation, *tinea corporis* was the predominant single clinical manifestation (n = 12, 40%) and frequently occurred alongside one (n = 10, 33.3%) or two (n = 1, 3.3%) additional lesions, confirming the role of *T. indotineae* in causing extensive and multifocal dermatophytosis [10]. As reported by Gupta et al. [3], in this study *tinea cruris* and *tinea faciei* were also observed as other common lesion sites. In addition, *T. indotineae* was associated with *tinea pedis* in four patients: in two cases as the sole clinical manifestation and in two cases in association with *tinea cruris*. In cases 1 and 2, the clinical presentation included onychomycosis, although nail involvement in *T. indotineae* infections is considered uncommon and has been rarely reported in the international literature [3,22].

The proportion of terbinafine-resistant strains obtained in this investigation was 60% (n = 18/30), similarly to those observed by Ribeiro dos Santos et al. in their previously mentioned study [5]. In particular, 14 of the 18 resistant strains (77.8%) were isolated from *tinea corporis*, either alone (n = 6, 33.3%) or in combination with other clinical manifestations (n = 8, 44.4%). These findings underscore the high pathogenic potential of *T. indotineae*, considering that in some patients the distribution of lesions involves much of the body surface.

As already discussed by the same [24] and other authors [25], cases 12 and 15 may be attributable to a sexually transmissible *T. indotineae* infection in men who have sex with men (MSM), which is a hypothesis supported by the clinical manifestations associated with *tinea cruris*. It is noteworthy that in 10 out of 13 patients with *tinea cruris*, *T. indotineae* showed resistance to terbinafine, including the two MSM cases. This potential transmission route should be considered in patients with sexual behaviors associated with a higher risk of sexually transmissible infections (STIs), in those presenting dermatophytosis lesions at anatomical sites commonly linked to close physical contact (e.g., genitals, groin, buttocks, or face) without an obvious alternative source of infection (such as animal exposure or concurrent foot/nail disease), and in cases where similar lesions are observed in sexual partners.

Molecular investigations identified L393S (n = 9) and F397L (n = 9) substitutions in the same proportion (50% each one) in the 18 resistant strains, confirming them among the most frequent single mutations in *SQLE* gene [3].

Decreasing sequencing costs, coupled with high-throughput capabilities and user-friendly bioinformatics platforms, have established NGS as a robust alternative for molecular typing. In this study, analysis was performed on three strains representing distinct resistance profiles: one terbinafine-susceptible strain and two terbinafine-resistant strains harboring the L393S and F397L mutations, respectively. The NGS results for these former samples were perfectly concordant with findings obtained via Sanger sequencing. Furthermore, the detection of the A448T mutation aligns with previous reports suggesting that this substitution independently confers resistance to azoles but results in susceptibility to terbinafine [3,4]. In contrast, terbinafine resistance is specifically associated with mutations at positions 393, 397 or 415, such as L393S, F397L and F415C, respectively [4]. NGS demonstrated high robustness even at a depth of 100 K reads. This capacity enables the simultaneous processing of a substantially higher number of samples per run—up to 200,000 using the P1 flow cell—consequently lowering costs.

In this study, the mRNA sequence MW187977.1 was used as the reference wild-type *SQLE* sequence, in accordance with previous reports [6,7,20]. Amplification and sequencing of the target DNA revealed a 62 bp Operon “insertion” within the VCF report (GTAAGCATATGTTTTCACTCCCTTTGTTACTGGTTAGTGGTTACTAACATCCTACTATGTAG). This feature can be managed either by modifying the laboratory protocol (e.g., by amplifying RNA) or by designating the sequence as a “control” during bioinformatic analysis, since it is consistently present in all DNA samples.

Given the critical need for rapid information to guide optimal pharmacological treatment and improve patient’s clinical conditions, the DermaGenius^®^ Resistance Multiplex real-time PCR assay was usually used as an initial routine screening step. Even if minimal inhibitory concentration (MIC) values are not available, genotypic data remain reliable, as they frequently correspond with the phenotypic resistance profile [5,26].

Therefore, molecular approaches are essential for accurately defining the geo-graphical distribution of *T. indotineae* and for enabling rapid and reliable diagnosis, thereby supporting appropriate therapeutic management in the context of increasing antifungal resistance. Considering that such advanced diagnostic techniques are not widely available in routine microbiology laboratories, the findings highlight the importance of specialized mycological reference centers. These laboratories play a pivotal role in improving diagnostic accuracy, preventing the underestimation of *T. indotineae* circulation in Italy, particularly with regard to antifungal resistance behavior.

## 5. Conclusions

This study provides further evidence of the emergence of *T. indotineae* in Italy and highlights the possible occurrence of local transmission beyond imported cases. The high proportion of terbinafine-resistant strains, mainly associated with L393S and F397L substitutions in the *SQLE* gene, confirms the growing clinical relevance of antifungal resistance in this species. NGS analysis, although preliminary, encourages the authors to extend this approach to the remaining strains in order to evaluate whether it could be incorporated into the routine diagnostic workflow. Considering that the NGS technique is currently considered the most reliable and advantageous in terms of time and throughput, it may also be useful for investigating terbinafine-susceptible strains to obtain additional information, such as the A448T mutation identified in this study. The predominance of extensive dermatophytosis, particularly *tinea corporis* which is often associated with additional lesions, underscores the pathogenic potential of this dermatophyte. Molecular diagnostic tools are essential for accurate species identification and the rapid detection of resistance-associated mutations, enabling appropriate therapeutic management. As these techniques are not widely available in routine laboratories, the availability of specialized mycological reference centers is crucial to prevent the underestimation of *T. indotineae* circulation and antifungal resistance in Italy.

## Data Availability

The original data presented in the study are openly available in the GenBank database under accession number listed in Table 1.

## Abbreviations

The following abbreviations are used in this manuscript:

INDEL	Insertion/deletion
ITS	Internal transcribed spacer
IZSUM	Istituto Zooprofilattico Sperimentale dell’Umbria e delle Marche “Togo Rosati”
MIC	Minimal inhibitory concentration
MSM	Men who have sex with men
NGS	Next-generation sequencing
PCR	Polymerase chain reaction
SNP	Single nucleotide polymorphism
SNV	Single nucleotide variant
*SQLE*	Squalene epoxidase gene
STI	Sexually transmissible infections
VCF	Variant Call Format
